# Differential patterns of disease and injury in Mozambique: New perspectives from a pragmatic, multicenter, surveillance study of 7809 emergency presentations

**DOI:** 10.1371/journal.pone.0219273

**Published:** 2019-07-10

**Authors:** Ana O. Mocumbi, Bonifácio Cebola, Artur Muloliwa, Frederico Sebastião, Samuel J. Sitefane, Naisa Manafe, Igor Dobe, Norberto Lumbandali, Ashley Keates, Nerolie Stickland, Yih-Kai Chan, Simon Stewart

**Affiliations:** 1 Instituto Nacional de Saúde, Maputo, Mozambique; 2 Universidade Eduardo Mondlane, Maputo, Mozambique; 3 Hospital Central da Beira, Beira, Mozambique; 4 Direcção Provincial de Sofala, Beira, Mozambique; 5 Hospital Central de Nampula, Nampula, Mozambique; 6 Hospital Geral de Mavalane, Maputo, Mozambique; 7 Australian Catholic University, Melbourne, Australia; UCIBIO-REQUIMTE, Faculty of Pharmacy, University of Porto, PORTUGAL

## Abstract

**Background:**

There is a paucity of primary data to understand the overall pattern of disease and injuries as well as related health-service utilization in resource-poor countries in Africa.

**Objective:**

To generate reliable and robust data describing the pattern of emergency presentations attributable to communicable disease (CD), non-communicable disease (NCD) and injuries in three different regions of Mozambique.

**Methods:**

We undertook a pragmatic, prospective, multicentre surveillance study of individuals (all ages) presenting to the emergency departments of three hospitals in Southern (Maputo), Central (Beira) and Northern (Nampula) Mozambique. During 24-hour surveillance in the seasonally distinct months of April and October 2016/2017, we recorded data on 7,809 participants randomly selected from 39,124 emergency presentations to the three participating hospitals. Applying a pragmatic surveillance protocol, data were prospectively collected on the demography, clinical history, medical profile and treatment of study participants.

**Findings:**

A total of 4,021 males and 3,788 (48.5%) females comprising 630 infants (8.1%), 2,070 children (26.5%), 1,009 adolescents (12.9%) and, 4,100 adults (52.5%) were studied. CD was the most common presentation (3,914 cases/50.1%) followed by NCD (1,963/25.1%) and injuries (1,932/24.7%). On an adjusted basis, CD was more prevalent in younger individuals (17.9±17.7 versus 26.6±19.2 years;p<0.001), females (51.7% versus 48.7%—OR 1.137, 95%CI 1.036–1.247;p = 0.007), the capital city of Maputo (59.6%) versus the more remote cities of Beira (42.8%—OR 0.532, 95%CI 0.476–0.594) and Nampula (45.8%—OR 0.538, 95%CI 0.480–0.603) and, during April (51.1% versus 49.3% for October—OR 1.142, 95%CI 1.041–1.253;p = 0.005). Conversely, NCD was progressively more prevalent in older individuals, females and in the regional city of Beira, whilst injuries were more prevalent in males (particularly adolescent/young men) and the northern city of Nampula. On a 24-hour basis, presentation patterns were unique to each hospital.

**Interpretation:**

Applying highly pragmatic surveillance methods suited to the low-resource setting of Mozambique, these unique data provide critical insights into the differential pattern of CD, NCD and injury. Consequently, they highlight specific health priorities across different regions and seasons in Southern Africa.

## Introduction

Africa faces a myriad of challenges to improve the quality of life and life-expectancy of more than one billion diverse peoples living on a vast continent [1]. These challenges will evolve in response to dynamic socio-economic changes when combined with sustained exposure to communicable diseases (CDs) such as HIV/AIDS, tuberculosis and malaria. New risks associated with urbanization of this region include adopting new occupations and modes of transport that expose people to high rates of injury [2, 3], as well as new lifestyles/behaviors that lead to non-communicable diseases (NCDs) in the longer-term [4]. In resource-poor settings, the lack of accurate health surveillance data devalues the relevance and achievability of aspirational goals for the African continent [5] and the provision of equitable, resilient and sustainable health care [6] in the face of multiple health care threats. Results from the 2016 Global Burden of Disease Study suggesting stagnant trends and even rising years lost to disability due to NCDs and injuries in sub-Saharan Africa [7, 8], highlight the need, more than ever, to direct limited public health and clinical resources to where they are most needed.

Contemporary studies have already confirmed a high burden of risk-factors for NCDs (including hypertension, energy-dense diets, sedentary behaviors and smoking) in poverty-stricken countries such as Malawi and Mozambique in Southern Africa [9, 10]. As reflected in the emergence of Type 2 diabetes [11] and hypertension-related stroke [12], in these vulnerable communities the future threat from NCD is substantive. Concurrently, the exposure to the risk of injury (especially among younger individuals) is a growing socio-economic threat [3, 13]. However, reflective of many other poverty-stricken countries with low-resources in sub-Saharan Africa [9, 10], there remains a paucity of primary data on the overall pattern of disease and injury to inform health-service priorities and planning; particularly as most health system initiatives specifically address the burden imposed by common forms of CD.

### Study objectives

In considering how to best close the knowledge gap in our knowledge of the overall pattern of disease and injury in Mozambique, we considered—**1**) the type and extent of health services available (noting a lack of ambulances and primary health care centers); **2**) a subsequent reliance on emergency departments at key regional hospitals; **3**) seasonal patterns of disease in vulnerable communities; and **4**) its dispersed and heterogeneous geographical regions, to develop a highly pragmatic stragety to capture reliable and robust data that could inform subsequent health care priorities and policy, as well as more definitive population surveillance studies in the future. Accordingly, we designed the MOZambique snApshot of emeRging Trends (MOZART) Disease Surveillance Study to document the characteristics and clinical spectrum of those seeking acute hospital care in the Northern, Central and Southern regions of Mozambique.

## Methods

### Study setting

**Fig 1:**
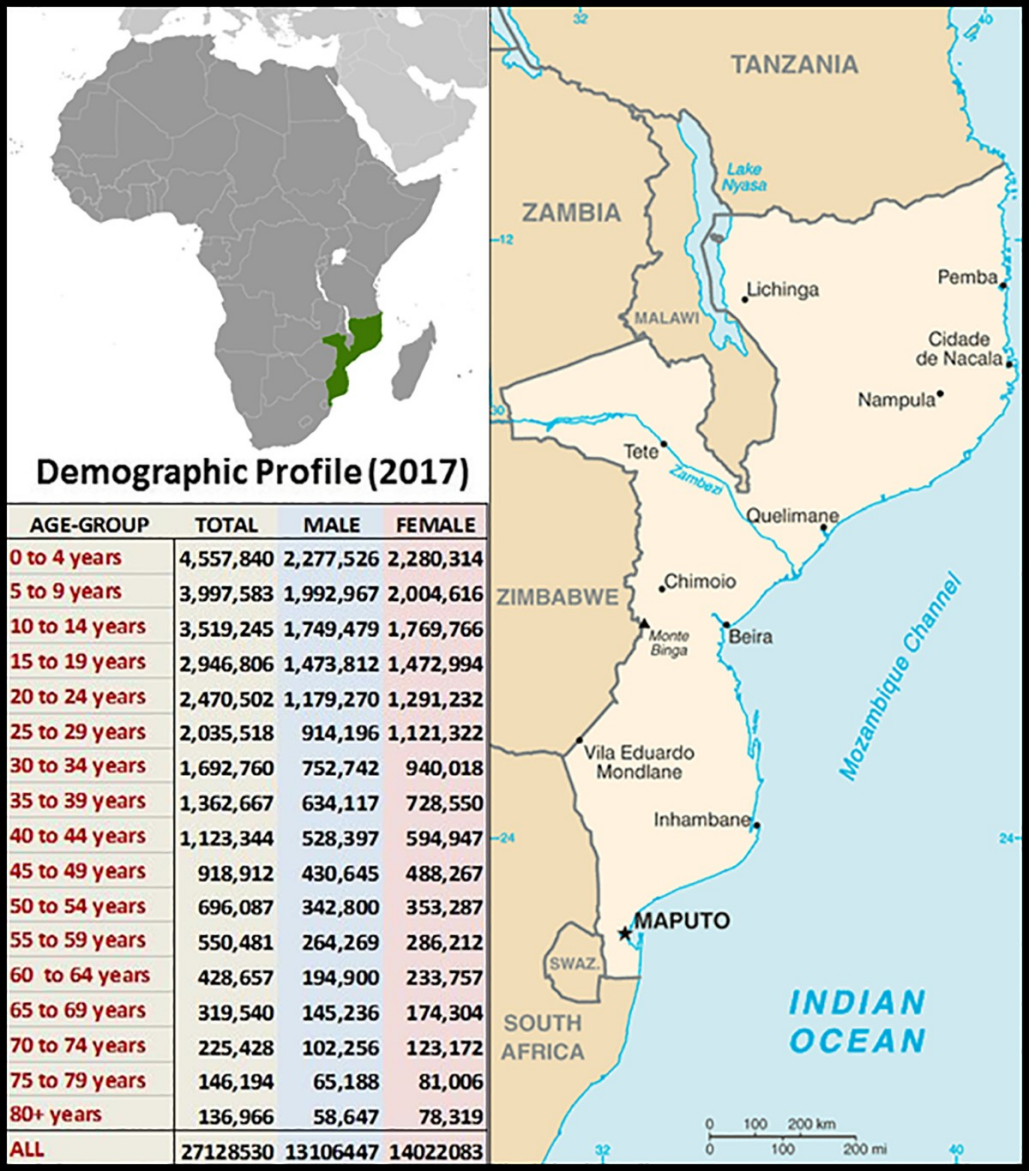
Geographic and demographic profile of Mozambique. Demographic profiling data (table inset) were sourced from http://www.ine.gov.mz/estatisticas/ (Accessed February 2019) and the public domain maps from https://www.drivingdirectionsandmaps.com/mozambique-google-map/ (Accessed May 2019).

Mozambique is a large and culturally diverse country located in South-East Africa (see **Fig 1**). With a rapidly growing but predominantly impoverished population of ~29 million (median age ~17 years), a life-expectancy expectancy below 60 years and, two-thirds living in rural/regional areas—like many other countries in Africa—Mozambique faces many socio-economic challenges. Almost the entire population is comprised of indigenous tribal groups, such as the Sena, Lomwe, Makhuwa and Tsonga; only 0.04% inhabitants identify as Euro-African, European and/or Indian. A recent study demonstrated that one in two households are deprived with a marked urban (29.3% poor) versus rural (59.3% poor) divide[14]. As in many low-income countries in the region, access to health care is sub-optimal. Beyond inter-hospital transfers there are no public ambulance services for acute injuries (including motor vehicle accidents), and the primary care routine services for NCDs are mainly directed to HIV care. As such, the national health system covers approximately 40% of the population, with a rate of 0.04 physicians and 0.07 hospital beds/1,000 persons[15]. The challenge of providing optimal health services is exacerbated by geography; Mozambique covers ~800,000 km^2^ with a coastline stretching ~2,500 km. The country also faces climatic challenges. With a Köppen Climate Classification of “Tropical Savanna”, with mean temperatures ≥23°C year-round and distinctive wet and warm versus dry season during the “cooler months” [16] the underlying risk of malaria and other CDs is high.

### Study design

MOZART was a prospective, hospital-based, multi-centered, surveillance study that conformed to STROBE guidelines for observational studies [17]. Ethics approval was granted by the Mozambican National Bioethical Committee/Ministry of Health, Mozambique and the study prospectively registered with the Australian and New Zealand Clinical Trials Registry—ACTRN12616001250426.

**Fig 2:**
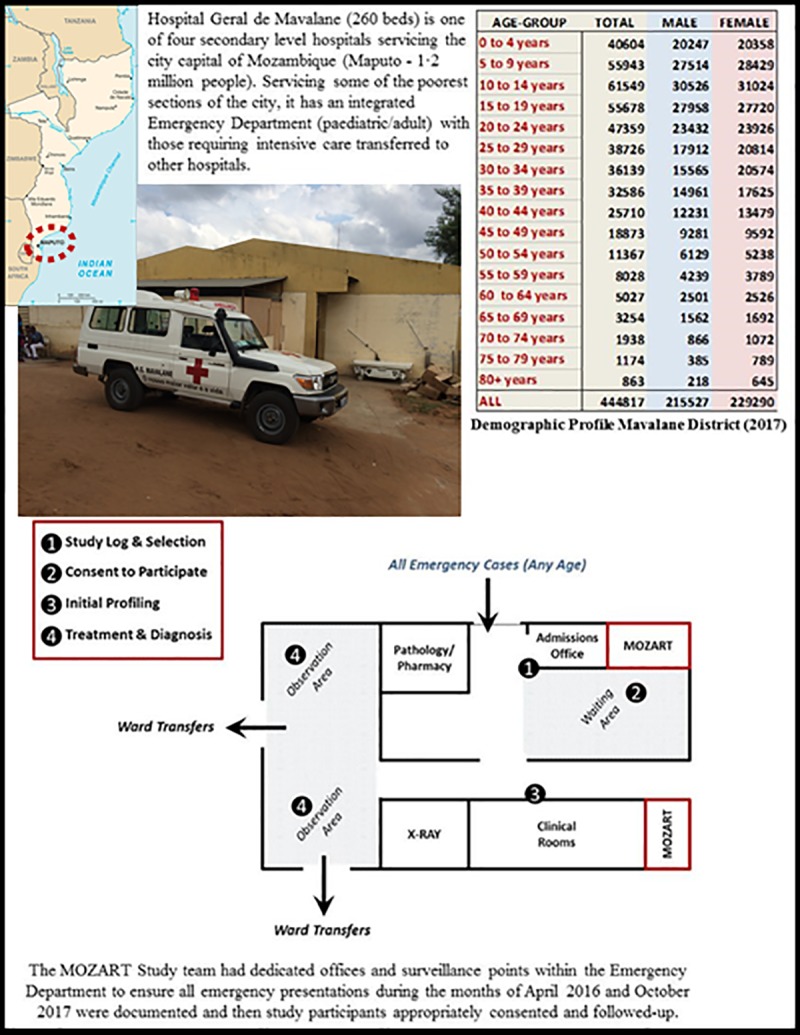
Hospital Geral de Mavalane (260 beds) in Maputo (Southern Mozambique). Public domain map of Mozambique sourced from: https://www.drivingdirectionsandmaps.com/mozambique-google-map/ (Accessed May 2019).

**Fig 3:**
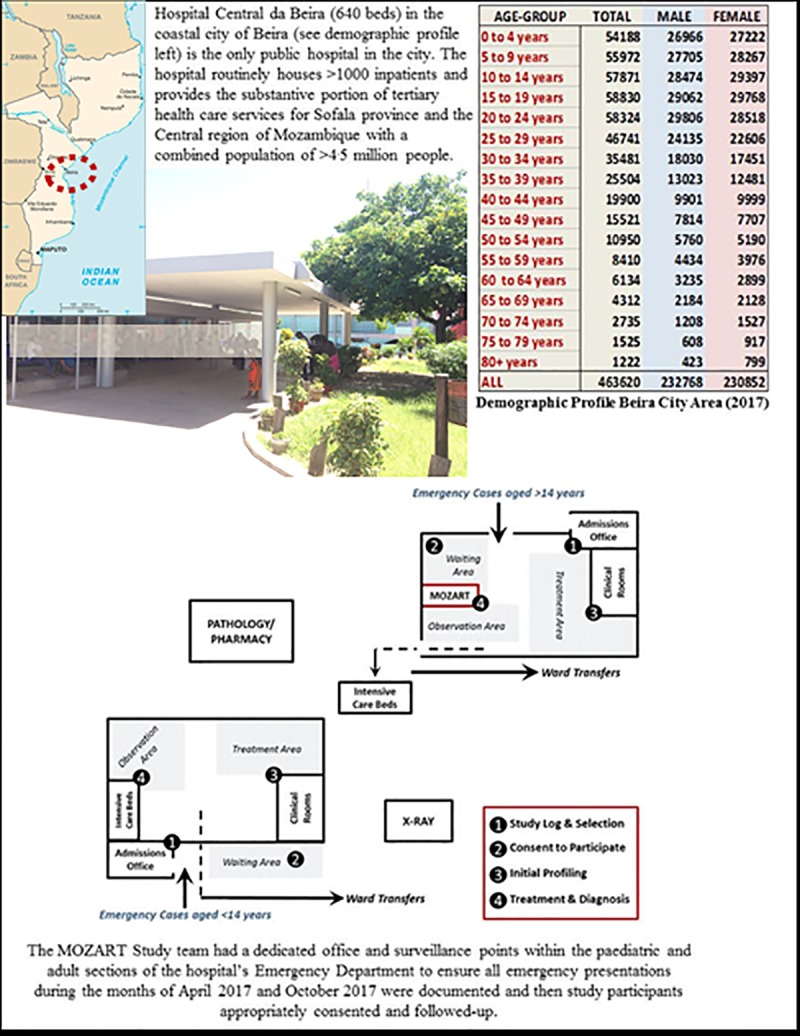
Hospital Central da Beira (640 beds) in Beira (Central Mozambique). Public domain map of Mozambique sourced from: https://www.drivingdirectionsandmaps.com/mozambique-google-map/ (Accessed May 2019).

**Fig 4:**
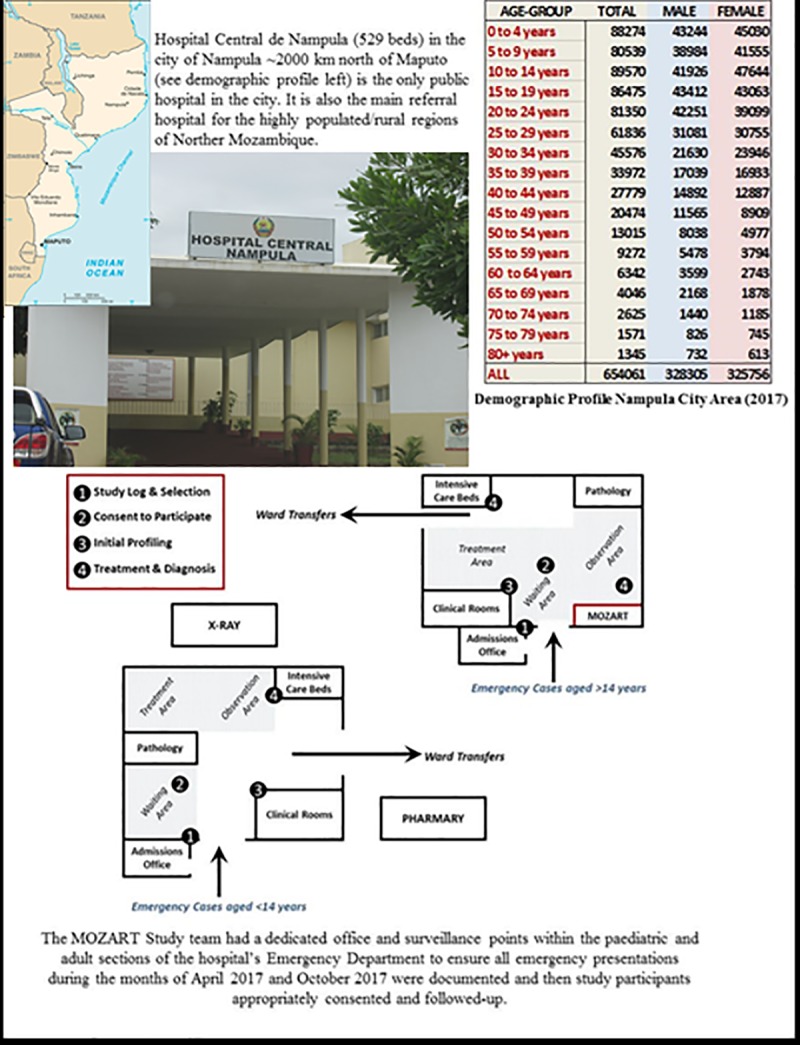
Hospital Central de Nampula (529 beds) in Nampula (Northern Mozambique). Public domain map of Mozambique sourced from: https://www.drivingdirectionsandmaps.com/mozambique-google-map/ (Accessed May 2019).

Following a highly essential pilot study in Maputo (Mozambique’s capital city) in April 2016, we completed an investigation of cases of all ages presenting to the emergency departments of three purposefully selected hospitals located in the Southern—Maputo (Hospital Geral de Mavalane–see **Fig 2**), Central—Beira (Hospital Central de Beira–see **Fig 3**), and Northern—Nampula (Hospital Central de Nampula–see **Fig 4**)–regions of Mozambique. The study was deliberately implemented in the two distinct seasons affecting Mozambique to account for seasonal influences on the pattern of disease; the typically drier and colder month of April versus the wetter and hotter month of October. As shown in **Figs 2–4**, these “sentinel” hospitals provide emergency health services to surrounding urban communities and have referrals from surrounding districts, since they are based in the three main cities in Southern, Central and Northern Mozambique. Critically for assessing the NCD burden, the emergency services in Beira and Nampula hospitals are the only entry point for public hospital admissions in their entire cities (excluding a dedicated psychiatric hospital in Nampula city). The hospital in Maputo provides near equivalent services to the largest urban district in the country’s capital, being one of its five first-referral hospitals. Because the referral system in the public sector in Mozambique requires that every patient admitted to hospital wards enter through the emergency departments, these services represent an important barometer of the burden of disease and service demand in their respective communities. Importantly, these emergency services are also entry points for patients with HIV, Malaria and Tuberculosis who need differentiated assessment, management or follow-up outside primary care facilities due to the severity of the disease, occurrence of complications or therapy failure.

Administrative, clinical and research staff at each hospital were engaged to operationalize the study protocol according to the physical characteristics of their emergency department(s). Medically qualified personnel from Mozambique’s National Institute of Health directly supervised study activities for the duration of each study period; providing first-line verification/review of each participant’s diagnosis following initial assessment/management.

### Study participants

Based on the previously observed caseload at each hospital, a feasible target of at least 1,000 patients per month per hospital was established; the initially set ratio of five patients to one participant being recruited was ultimately achieved. The age and gender of every individual presenting to each emergency department during each surveillance period were entered into a study logbook and used to randomly select patients for study participation. Following the unexpected introduction of a “service fee” for emergency cases in Beira and Nampula just prior to October 2017, recruitment rates were increased to three to one in October 2017 for Nampula. Informed consent was obtained from all participants or their legal guardians by a medically qualified researcher. Each participant was given a unique identifier to protect their anonymity and only de-identified data were used to generate study findings.

### Study data

All study data were collected using standardized methods and case-report forms (see S1 File). Based on pilot testing, data were collected via a combination of fixed and open-ended questions to obtain information on each participants’ demography, past medical history, presenting complaint, clinical profile, clinical investigations, in-hospital treatment, location of injury (where appropriate) and outcome following emergency treatment. Every case was reviewed by a medically qualified supervisor and classified (up to 3 codes) according ICD 10 coding [18]. These data were initially collected on paper-based case-report forms that were then verified and entered into a purpose-built database (Microsoft Access) by study supervisors using secure/password-protected, study lap-tops. De-identified data were then subject to a rigorous protocol of data cleaning (with rapid data queries for each team) and verification to build the MOZART Master Database. Final ICD 10 coding was performed, using a consensus approach, by AM and SS who also assigned each presentation attributable to a CD, NCD or injury.

### Study outcomes

As described above, the MOZART Study was specifically designed to provide critical insights into the pattern of disease and injury in Mozambique via one of the major points of interaction between those affected and the health care system. The primary outcome of interest (reported on an age and sex-specific basis) was the observed number and proportion (relative to the total number of emergency case presentations) classified as CD, NCD or physical injury at each hospital during the study periods. Secondary outcomes of interest included—**1**) the timing of case-presentations by hour of the day; **2**) pattern of presentations according to surveillance site and season (April versus October); and **3**) based on the random selection of cases (with minimal evidence of selection bias), the indicative, annual burden/pattern of disease and injury at each hospital.

### Statistical analyses

A minimum of 2,000 cases per site, provided robust data to investigate each outcome of interest; the 95% confidence intervals (CI’s) for any diagnostic grouping occurring in >5% of cases being ± 1.35%. All study findings were examined in aggregate and according to a participant’s age, gender, hospital location and month of presentation as outlined in a pre-specified Statistical Analysis Plan (*available on request*). Accordingly, participants were categorized as infants (aged <12 months), children (aged 1–9 years), adolescents (aged 10–19 years) or adults (≥20 years). Discrete and continuous data are presented as a count with proportion and mean (± standard deviation) or median (interquartile range [IQR]), respectively, with standard methods for group comparison. Multiple logistic regression (entry model) was used to explore the correlates (presented as adjusted odds ratios [OR] with 95% CI) of CD, NCD and injury cases according to age, gender, location and presentation month. Time of presentation (24-hour clock) is presented as descriptive data only. All study data were analyzed using SPSS for Windows Version 24.0 (SPSS Inc, Chicago, Illinois).

## Results

**Fig 5:**
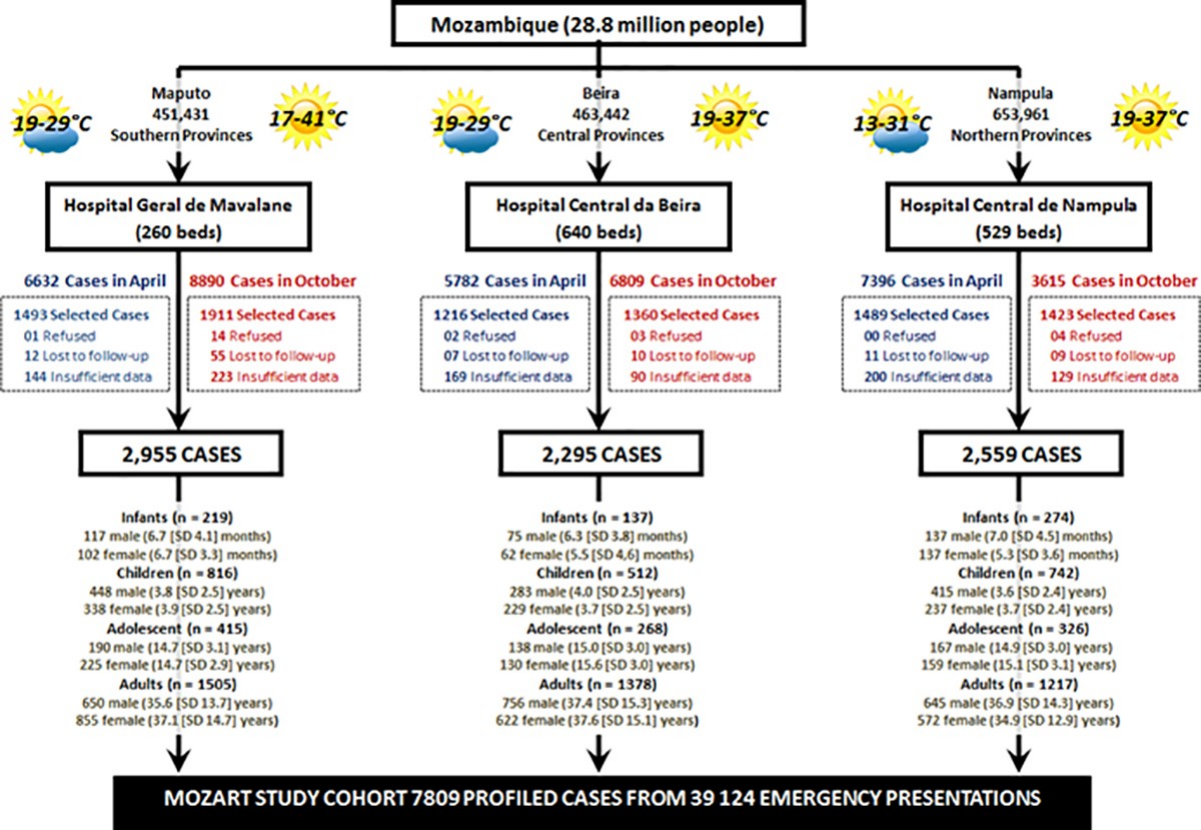
Overview of the MOZART Study cohort. Randomly selected cases without data comprised 10.5%/15.3% of cases in Maputo, 14.6%/7.8% of cases in Beira & 14.2%/10.0% in Nampula for the April/October survey periods. Historical weather data from: https://www.timeanddate.com/weather/mozambique (accessed May 2018).

A total of 39,124 individuals of all ages sought emergency care at the three participating hospitals during the study period—see **Fig 5**. Following participant refusal (21 cases) combined with lack-of-follow-up or insufficient clinical data, a total of 7,809/8,892 selected cases (87.8%) form the study cohort–**Table 1** summarizes the socio-demographic and clinical profile per age group.

**Table 1:** Cohort profile according to age and sex.

	630 Infants Aged ≤12 months		2,070 Children Aged 1–9 years		1,009 Adolescents Aged 10–19 years		4,100 Adults Aged ≥20 years	
	Male (n = 329)	Female (n = 301)	Male (n = 1,146)	Female (n = 924)	Male (n = 495)	Female (n = 514)	Male (n = 2,051)	Female (n = 2,049)
***Demographic Profile (all cases)***								
Age, mean (± SD), months* & years	6.7 ± 4.2 *	5.8 ± 3.8 *	3.8 ± 2.5	3.8 ± 2.5	14.9 ± 3.0	15.1 ± 3.0	36.7 ± 14.5	36.7 ± 14.3
African ancestry (%)	325 (98.8%)	295 (98.0%)	1,139 (99.4%)	915 (99.0%)	493 (99.8%)	513 (99.8%%)	2,038 (99.4%)	2,039 (99.5%)
***Medical History (n = cases where data were self-reported and deemed reliable)***								
Hypertension (%) n = 6,524	-	-	-	-	-	-	500 (28.5%)	683 (38.3%)
Passive/Active Smoker (%) n = 6,524	26 (9.7%)	23 (9.2%)	113 (12.1%)	79 (10.8%)	62 (15.9%)	43 (10.3%)	388 (22.1%)	218 (12.2%)
HIV/AIDS (%) n = 6,523	35 (13.1%)	20 (8.0%)	95 (10.2%)	75 (10.2%)	29 (7.5%)	35 (8.4%)	226 (12.9%)	336 (18.9%)
Tuberculosis (%) n = 7,046	4 (1.3%)	6 (2.2%)	32 (2.9%)	31 (3.6%)	13 (2.8%)	20 (4.1%)	142 (7.2%)	144 (7.3%)
Malaria (%) n = 7,438	65 (21.0%)	54 (19.6%)	465 (42.8%)	333 (38.5%)	268 (58.0%)	264 (53.5%)	1,358 (68.8%)	1,315 (66.8%)
Anemia (%) n = 6,524	28 (10.5%)	20 (8.0%)	89 (9.6%)	70 (9.6%)	32 (8.2%)	57 (13.6%)	168 (9.6%)	340 (19.1%)
Chronic Diarrhea (%) n = 7,438	4 (1.5%)	3 (1.2%)	26 (2.8%)	12 (1.6%)	8 (2.1%)	6 (1.4%)	43 (2.5%)	45 (2.5%)
Respiratory Disease (%) n = 7,438	45 (14.5%)	39 (14.2%)	209 (19.2%)	155 (17.9%)	61 (13.2%)	68 (13.8%)	238 (12.1%)	250 (12.7%)
Diabetes (%) n = 6524	-	-	65 (7.0%)	66 (9.0%)	25 (6.4%)	32 (7.6%)	100 (5.7%)	127 (7.1%)
Prior Injury/Trauma (%) n = 1,336	1 (1.4%)	0 (0%)	4 (2.0%)	8 (5.4%)	11 (13.9%)	8 (11.1%)	60 (19.5%)	40 (10.4%)
Cardiovascular Disease (%) n = 6,101	2 (0.8%)	1 (0.4%)	7 (0.8%)	1 (0.1%)	3 (0.8%)	4 (1.0%)	30 (1.8%)	39 (2.5%)
Use Traditional Medicines (%) n = 6,524	59 (22.0%)	56 (22.5%)	240 (25.8%)	185 (25.2%)	64 (16.5%)	77 (18.4%)	358 (20.4%)	374 (21.0%)
***Clinical Presentation (n = cases where data were available/recorded)***								
BMI, mean (SD), kg/m^2^ n = 7,519	-	-	-	-	19.7 ± 7.8	20.3 ± 4.4	22.8 ± 4.8	25.1 ± 6.7
SBP/DBP, mean (± SD), mmHg n = 6,059	103 ± 23/ 68 ± 18	103 ± 22/ 70(18)	104 ± 17/ 69 ± 15	104 ± 16/ 70 ± 15	118 ± 18/ 72 ± 11	114 ± 16/ 73 ± 12	130 ± 22/ 81 ± 15	127 ± 15/ 82 ± 16
Heart Rate, mean (± SD), n = 7,652	126 ± 31	128 ± 30	117 ± 26	119 ± 27	95 ± 21	100 ± 21	86 ± 19	92 ± 19
Febrile—≥37.0 Celsius (%) n = 7,753	172 (52.4%)	141 (47.5%)	542 (47.8%)	424 (46.2%)	191 (38.7%)	192 (37.6%)	503 (24.7%)	571 (28.1%)
Hypoxic—0_2_ Sat <90% (%) n = 7,359	38 (12.9%)	42 (16.3%)	88 (8.2%)	73 (8.4%)	21 (4.4%)	14 (2.9%)	50 (2.6%)	56 (2.9%)
***Cause of Emergency Presentation (all cases)***								
Communicable Disease (%)	240 (72.9%)	221 (73.4%)	717 (62.6%)	608 (65.8%)	212 (42.8%)	257 (50.0%)	787 (38.4%)	872 (42.6%)
Non-Communicable Disease (%)	64 (19.5%)	55 (18.3%)	126 (11.0%)	100 (10.8%)	73 (14.7%)	143 (27.8%)	632 (30.8%)	770 (37.6%)
Injury/Trauma (%)	25 (7.6%)	25 (8.3%)	303 (26.4%)	216 (23.4%)	210 (42.4%)	114 (22.2%)	632 (30.8%)	407 (19.9%)

Footnote

* Age is provided in months for Infants

### Cohort profile

Overall, the study cohort included 4,021 males and 3,788 (48.5%) females and comprised 630 infants (8.1%), 2,070 children (26.5%), 1,009 adolescents (12.9%) and, 4,100 adults (52.5%). Previous history of CD (most notably malaria and HIV/AIDS) was high across all age groups. Contrastingly, levels of pre-diagnosed NCD were relatively low. However, future risk levels (with age and gender-based differentials) for NCD were noticeably high. Although there was minimal obesity among younger individuals (<4% in adolescents), 396 adult women (19.9%) were assessed as obese compared to 143 males (7.1%)—OR 3.24 (95% CI 2.65–3.97); p<0.001. Alternatively, 218 adult women (12.2%) versus 388 adult men (22.1%), had a history of smoking—OR 0.491 (95% CI 0.410–0.589); p<0 .001. Moreover, all ages were exposed to a combination of burning coal (86.8%) and/or wood (10.1%) used for cooking/heating purposes in their home.

### Health outcomes

Following initial assessment and management, a total of 31 (0.40%, 95% CI 0.26–0.54%) participants had died in the emergency department; 1576 (20.2%, 95% CI 19.3 to 21.1%) were formally admitted to hospital wards; a further 50 (0.64%, 95% CI 0.46–0.82%) cases in Maputo were transferred to the country’s main tertiary-referral hospital.

### Diagnostic profile

**Fig 6:**
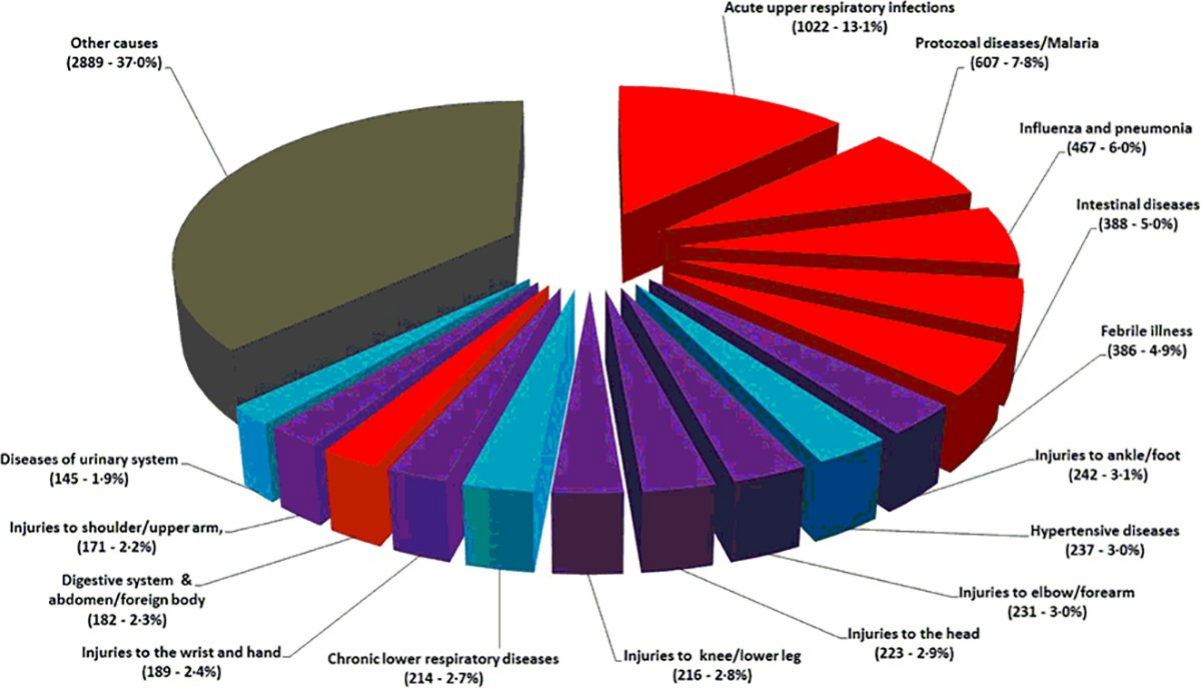
Top 15 diagnoses in the MOZART cohort (representing 63% of all diagnoses)–ICD10 coding. Red segments = CD, Blue segments = NCD, Purple segments = Injury and Grey segment = other diagnoses comprising CD, NCD and Injury.

**Fig 7:**
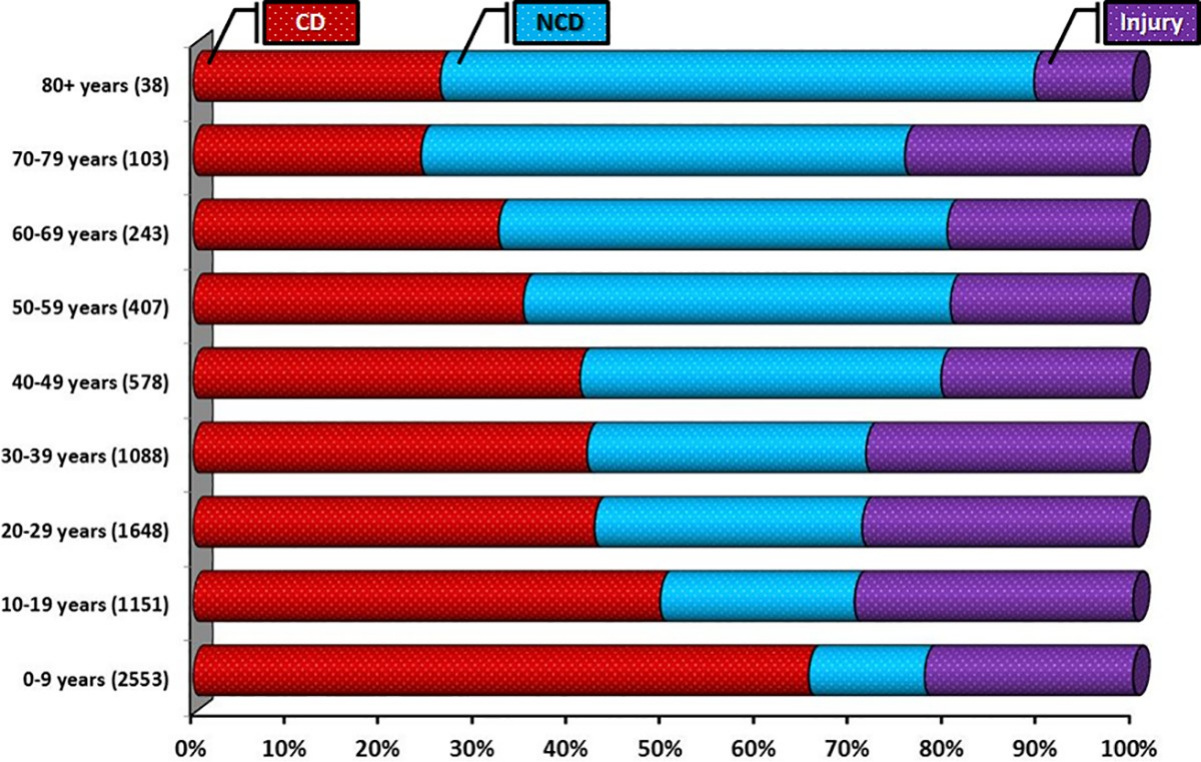
Pattern of CD, NCD and injury according to age profile.

Overall, 3914 (50.1%, 95% CI 49.0–51.2%) individuals presented with a CD; the remainder of cases being attributable to NCD (1,963 [25.1%], 95% CI 24.2–26.1%) or injury (1932 [24.7%], 95% CI 23.8–25.7%)–see **Fig 6**. The gender balances in each presentation category were broadly equivalent except for adolescents (p<0.001) in whom there was a greater proportion of injuries among adolescent males. Alternatively, there was a marked gradient in the balance of CD versus NCD cases according to increasing age—see **Fig 7**. The top 15 diagnoses (ICD 10 coding) accounted for 63% of cases overall; with the top five diagnoses, including acute respiratory infections, malaria and influenza/pneumonia, all being forms of CD.

#### Communicable diseases

**Fig 8:**
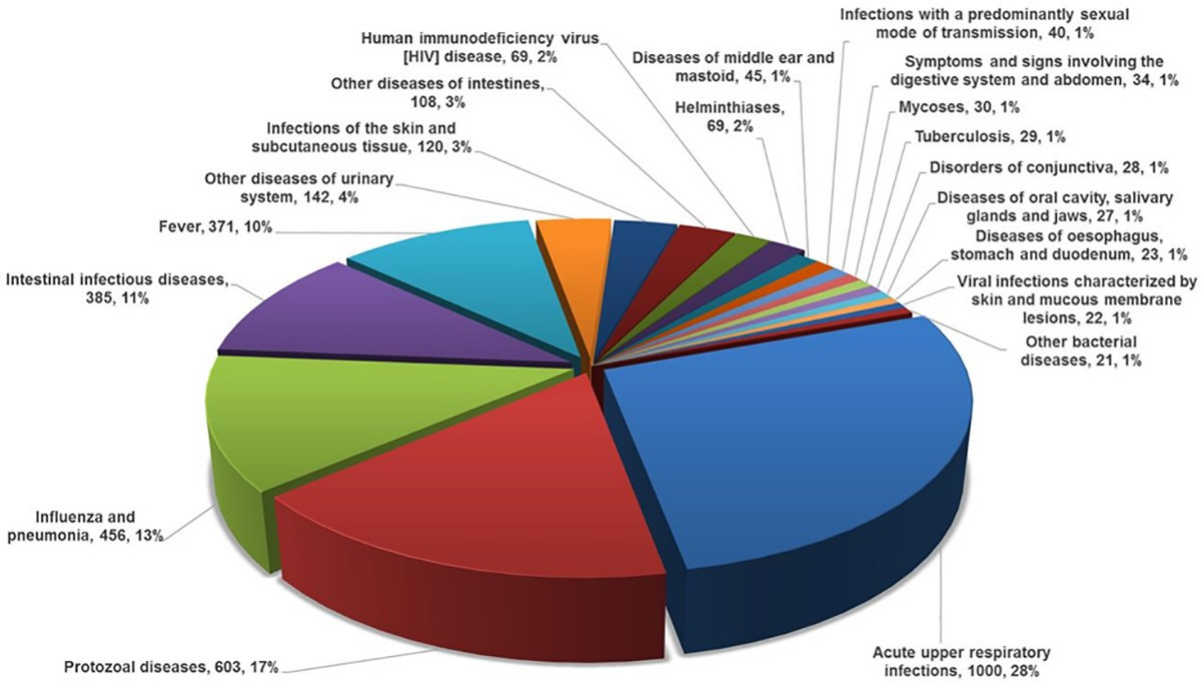
Twenty most common forms of communicable disease–ICD 10 coding (3,622 Cases).

Those presenting with a CD (see **Fig 8**) were more likely to be younger (mean age 17.9±17.7 versus 26.6±9.2 years, adjusted OR 0.974 95% CI 0.972–0.977; p<0.001), female (51.7% versus 48.7%, adjusted OR 1.137, 95% CI 1.036–1.247; p = 0.007) and, present in Maputo (59.6% of cases) compared to Beira (42.8%, adjusted OR 0.532, 95% CI 0.476–0.594) and Nampula (45.8%, adjusted OR 0.538, 95% CI 0.480–0.603). The latter difference was largely driven by more presentations of acute upper respiratory infections in Maputo (615 versus 407 cases combined for Nampula/Beira).

#### Non-communicable diseases

**Fig 9:**
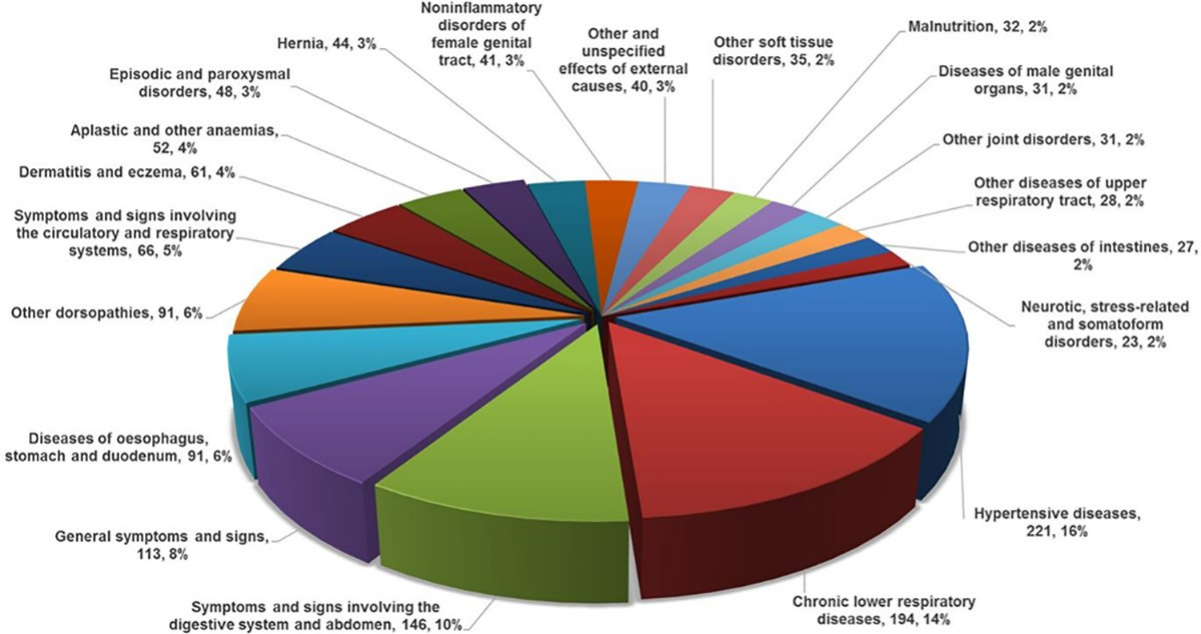
Twenty most common forms of non-communicable disease–ICD 10 coding (1,415 Cases).

The most common NCD-related presentations were hypertension (221 cases), chronic respiratory disorders including asthma (194 cases) and urogenital disorders (145 cases)–see **Fig 9**. NCD cases were more likely to be relatively older (mean age 30.8±20.4 versus 19.3±17.5 years, adjusted OR 1.031, 95% CI 1.028–1.034; p<0.001), female (28.2% versus 22.3%, adjusted OR 1.381, 95% CI 1.241–1.619; p<0.001) and more present in Beira (30.5% of cases) compared to Nampula (23.3%, adjusted OR 0.798, 95% CI 0.699–0.912; p<0.0001) and Maputo (22.6%, adjusted OR 0.703, 95% CI 0.618–0.800; p<0.001).

#### Injuries

**Fig 10:**
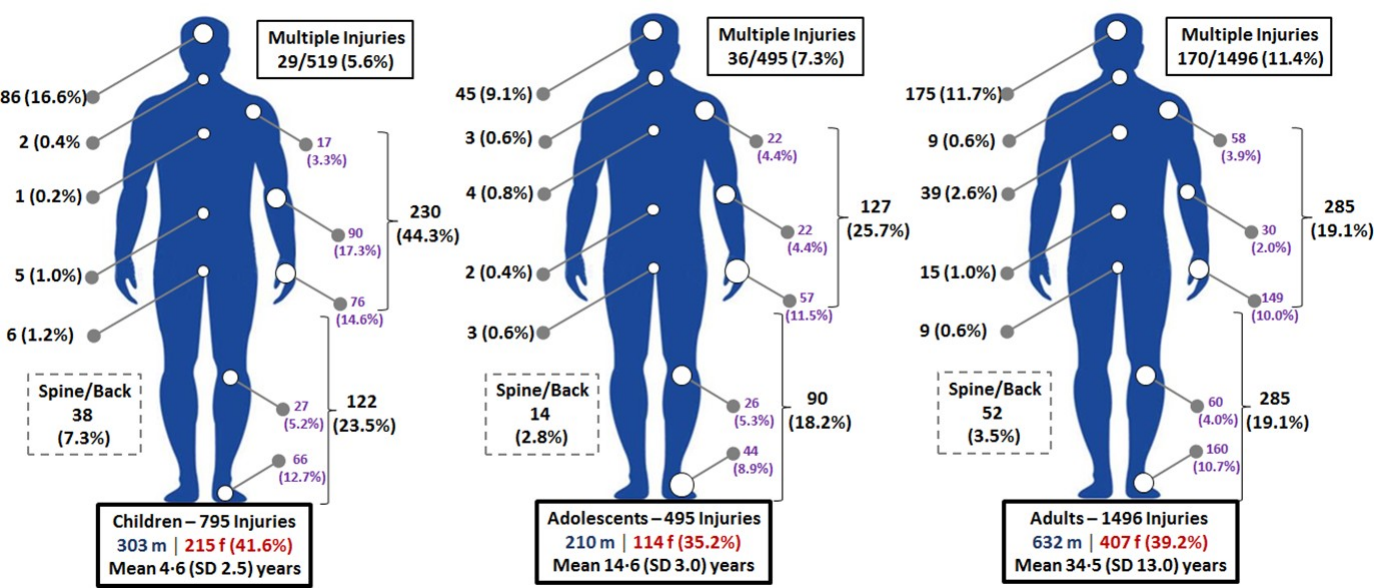
Location of injuries in children (795 injuries), adolescents (495 injuries) and adults (1,496 injuries).

Isolated injuries to the arms and legs along with injuries to the head were common in all age groups–see **Fig 10**. Alternatively, the proportion of multiple injuries/trauma increased with age (>10% absolute difference between children and adults), whilst ingestion of foreign bodies (95 cases overall) were more common in infants and children. Injury cases had a similar age profile to the rest (mean age 22.3±16.8 versus 22.2±19.6 years; p = 0.520), but were more likely to be male (29.1% versus 20.1%—adjusted OR 1.591, 95% CI 1.432–1.769; p<0.001) and present in Nampula (30.1% of cases) compared to Beira (26.8%, adjusted OR 0.805, 95% CI 0.710–0.914; p<0.001) and Maputo (17.8%, adjusted OR 0.493, 95% CI 0.434–0.559; p<0.001).

### Timing of emergency presentations

**Fig 11:**
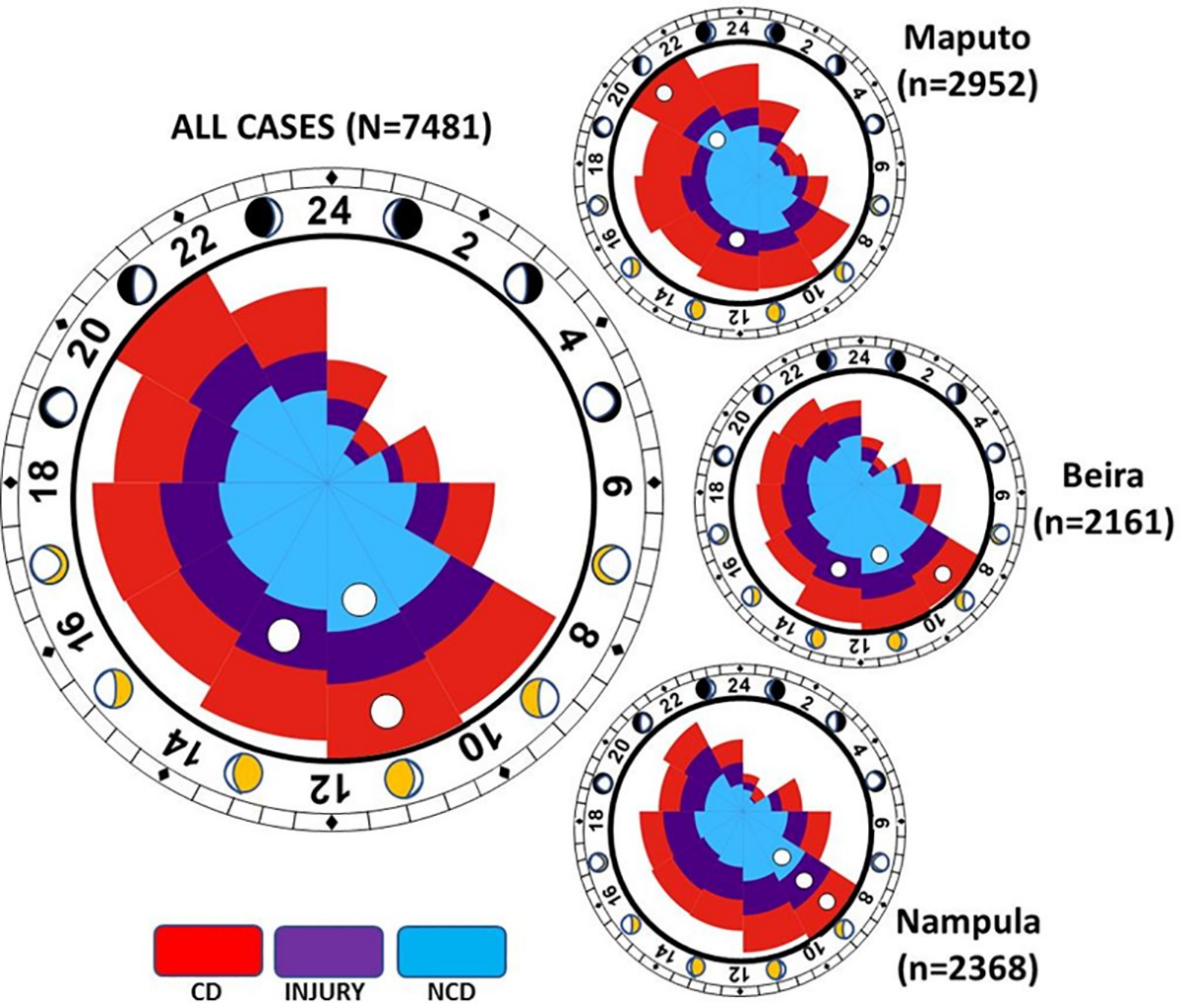
24-hour distribution of emergency presentations according to type. Each circle represents the 24-hour clock and the wedges the relative volume of cases per 2-hour period. The peak volumes in cases are indicated by the white circle for each category of presentation.

The overall pattern and volume of hospital emergency presentations was not uniform; both from a 24-hour and a seasonal perspective (noting preceding changes in emergency services fees in Beira and Nampula). With notable differences for each region (including a peak in CD cases in Maputo), collectively, case-presentations increased markedly from 8am onwards with peak activity levels around noon for CD and NCD cases and early afternoon for injuries—see **Fig 11**. There were also variations in the volume and composition of presentations between April and October. In Maputo and Beira there were 2,258 and 1,027 more cases, respectively, in October. On an adjusted basis, CD cases were more likely to present in April (51.1% versus 49.3%—adjusted OR 1.142, 95% CI 1.041–1.253; p = 0.005). Alternatively, injury/trauma cases were (borderline significance) more common in October (25.4% versus 24.0%; adjusted OR 1.109, 95% CI 0.999–1.231; p = 0.053). No such difference was found in respect to NCD (25.0% in April versus 25.0% in October; p = 0.183).

## Discussion

To our knowledge, these data are unique to Mozambique, Southern Africa and indeed wider sub-Saharan Africa. Whilst not definitive in capturing the epidemiological pattern of disease and injury in Mozambique, they capture an important facet of the burden they impose on a key health service in that country–emergency care provided by regional hospitals. As such, they complement the findings of broader risk surveillance studies [9, 10] and burden of disease reports for Africa [19] by describing (in detail) the overall—and then differential—pattern of hospital presentations with CD, NCD and injuries in three distinct regions of Mozambique during two distinct climatic seasons and across a 24-hour period. Servicing a predominantly young and deprived local population, with limited resources, all three participating hospitals contend with 50–100,000 emergency cases per annum. Overall, we found that CD continues to generate one half of these emergency cases. As in other impoverished African countries [20], fever was a common trigger-point for seeking acute care for infants and children, notably in the evenings. NCD and injury were evenly balanced in the remainder of our cohort. However, the predominance of CD declined with age, with NCD predominant in (relative to CD cases) older age groups (men and women). There were also important differentials in the pattern of injuries according to the gender and age of affected individuals. From a health service and continuum of care planning perspective, “peaks” and “troughs” in the volume and nature of cases on both a 24-hour and seasonal basis further complicate these observed differentials.

It is important to emphasize that to date, there are only fragmented data to describe the overall burden and pattern of disease and injury in Mozambique and many other African countries. This situation is reflected in a reliance on modelling for burden of disease reports [8, 19] and the lack of surveillance programs for NCDs and injuries in most countries [13]. Alternatively, programs focusing on CD surveillance remain relatively well-funded. In response, we selected three “sentinel” sites in Mozambique to generate valid and representative health surveillance data, adapted to a low-resource context that could inform the provision of scarce health resources. On this basis, in recognition of the value of study findings, a detailed report on the MOZART Study findings has been already submitted to the Mozambique government and Department of Health to support future health planning.

While more sophisticated surveillance methods can be readily applied in high- (and indeed middle-) income countries to monitor the health of the population, including longitudinal cohort studies and electronic health surveillance, they are not feasible in very low-resource settings. It is critical therefore, to identify key points of health service interaction. Accordingly, emergency departments in poor countries like Mozambique are often the commonest entry points for disease and injury due to low availability of trained specialists and/or structured 24-hour services in primary care facilities. Notwithstanding low access to health care for remote populations and severe cases of trauma who die in the pre-hospital setting [13], therefore, they provide an important barometer of disease and injury within these vulnerable populations. As such, they may indeed be the only reliable point (using pre-existing infrastructure and trained personnel experienced in diagnosing complex cases) to apply good quality NCD and injury surveillance. In recognition of our pragmatic and now proven surveillance approach to generate unique and valuable findings in a resource-poor context, plans to replicate and expand our pragmatic model of surveillance to other African countries are well-advanced.

Pending detailed analyses of the individual contributors to CD, NCD and injury cases, a number of findings need comment. Firstly, we were able to confirm a substantive burden and history of CD in adults with many incident CD presentations among infants, children and adolescents. This is particularly true for malaria; now reported to be second only to HIV/AIDS in driving premature mortality, but currently in decline [21]. Along with drug-resistant tuberculosis [22] (in likely contrast to the control of HIV/AIDS [23]) malaria continues to represent a substantive health threat–in both hot/rainy as well as cool/dry seasons—and better therapeutic options for its control are being tested [24]. Interestingly, our findings also confirm the emerging threat of NCDs in countries like Mozambique; particularly when associated with migration to urban centers. As suggested, those individuals living to a relatively older age in Mozambique are not only more likely to develop a NCD requiring hospital care, but are also typically affected by multimorbid CD and NCD. Although lower respiratory infections are reportedly in decline globally [19], in accordance with community-based studies that shows very high usage of biomass fuels in both rural and urban areas in Mozambique [25], we found high exposure levels to biomass fuels revealing a latent risk for chronic obstructive pulmonary disease [26] and other chronic diseases such as asthma and cancer [27]. Consistent with an earlier report [2], we confirmed that a predominance of motor vehicle accident and violence-related injuries is not only prevalent in Maputo but also endemic to regional areas of Mozambique. The 24-hour pattern of injury presentations might be indicative of a resource-poor health system that engenders “survival of the fittest” and reliance on self-arranged prehospital transport to reach emergency care, but other factors may also be playing a role. In India prehospital transport practices showed that despite availability of free ambulance services for the public and private sectors people often preferred a locally available mode of transport like the auto-rickshaw, because most ambulances didn't follow protocols in terms of equipment or personnel, and none had coordination with local police [28]. Our data on the 24-hour pattern of emergency admissions differed from that found for admissions to intensive care unit in a community teaching hospital from a high income country, where 43.3% of the total admissions occurred afterhours—of which 55% were during the nighttime and 45% during the late-night hours [29]. Although there is a paucity of data describing seasonal patterns of illness in Africa [30], our data clearly demonstrates that this phenomenon (particularly in respect to CD and to some extent injuries) exists in the tropical climate of Mozambique [17].

Our study provides new insights into the pattern of disease and injury in a low-income African country that facilitate prioritization of care on a more individualized basis and with consideration of local context. This encompasses focusing on segments of the population traditionally neglected by health systems in Africa, such as adolescents and men. As in neighboring South Africa, due to unstructured transition of care management from pediatric to adult services, poor availability of integrated adolescent-friendly services and lack of functional school-based health programs, adolescents (particularly males) are frequently disenfranchised from a health perspective [31]. Considering the high burden of NCD and injuries among adolescents and adult men, new preventive programs targeting these neglected groups need to be explored and tested, including models offered by community health workers [32]. The highest death rates of adolescents in 2015 occurred in South Asia and sub-Saharan Africa [33]. In African low-to-middle income countries, death rates are nearly 13 times as high as those in high-income countries in adolescents aged 10–14 years, and over 7 times in adolescents aged 15–19 years [34]. Road injury is by far the leading cause of adolescent boys' mortality, accounting for about a quarter of all deaths in male adolescents aged 15–19 and being the third leading cause of adolescent girls' deaths in 2016, after maternal conditions and self-harm. African countries should carefully evaluate which specific elements of adolescent health should be monitored, and advocate towards shared investments in conditions affecting predominantly adolescents, to ensure a healthy productive population that can drive economic development. These insightful data also highlight the return for investment in applying mixed models of disease surveillance (including STEPS, affordable systems for routine data collection and comprehensive “snapshot” studies) and training health personnel to become self-sufficient in undertaking surveillance, something we achieved with the MOZART Study.

### Limitations

This study has a number of limitations that largely reflect the low-resource setting in which it was conducted. Firstly, with no ambulance services, it is most likely that many fatal injuries (including remote traffic accidents) and other debilitating conditions would not have reached hospital to be captured by our study. As highlighted by the high levels of reported use of traditional healers/medicines, it is also likely that many people with CD, NCD and minor injuries would have sought treatment in their local community. With no electronic records, it is also difficult to track individual participants beyond their immediate emergency care; something that would undoubtedly reveal poor health outcomes in the longer-term. As such, we are currently planning an extension of our surveillance methods to incorporate such follow-up. Finally, we do not present these data as a definitive and representative burden of disease study for Mozambique. However, considering the lack of concrete data informing major health reports for the region, our data provide important insights into the overall pattern of disease and injury in Mozambique (particularly from a health services perspective). They also present important caveats in considering other reports within sub-Saharan Africa that may not have accounted for important variances in the location and timing of surveillance on the pattern of disease and injury.

## Conclusions

The MOZART Study provides important and unique insights into the differential pattern of NCD, CD and injuries in Mozambique from multiple perspectives; these include the antecedents of disease and injury in vulnerable communities, patterns of access to limited health care resources and the potential influence of seasonal climate change. It highlights the continued vulnerability of the poorest peoples in Africa to chronic ill-health and/or premature death attributable to a broader and more complex range of CD, NCD and injuries than typically conveyed by burden of disease reports relying upon secondary sources of data. This includes high exposure to CD states such as malaria, but also the risk of chronic diseases likely linked to hypertension, indoor air pollution and smoke, as well as lack of access to immediate emergency assistance if they sustain a major injury.

## Supporting information

S1 Table Cohort profile according to age and sex. (XLSX)

S1 File Case report forms used for the study. (DOCX)
