# Supplementary material for: Differential patterns of disease and injury in Mozambique: New perspectives from a pragmatic, multicenter, surveillance study of 7809 emergency presentations
Source: PLoS One. 2019 Jul 10;14(7):e0219273. doi: 10.1371/journal.pone.0219273 (PMC6619685; doi:10.1371/journal.pone.0219273)
Supplement: S1 File — (DOCX) [file pone.0219273.s002.docx]

**Case Report Form – Registration Section (separated from MOZART Master Database)**

**
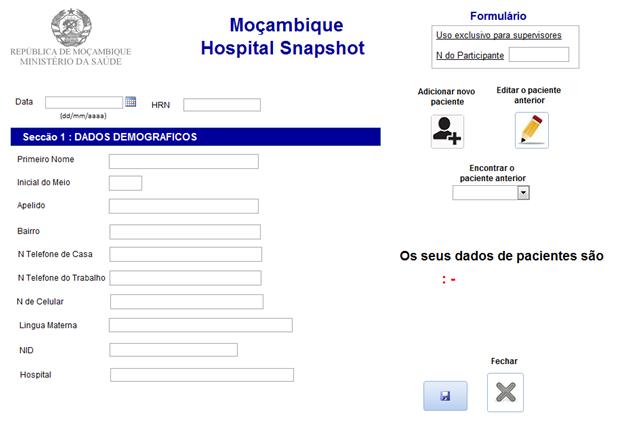
**

**Case Report Form – Page 1**

**
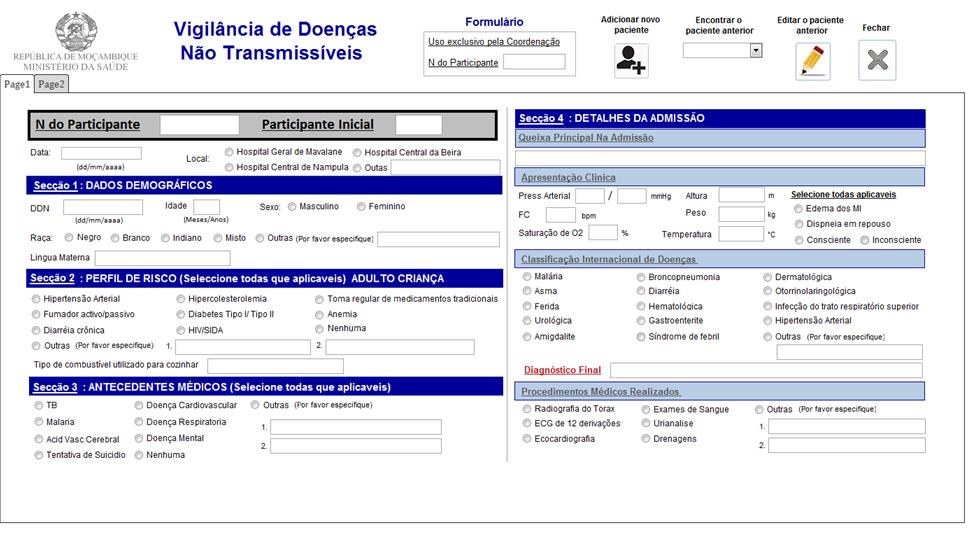
**

**Case Report Form – Page 2**

**
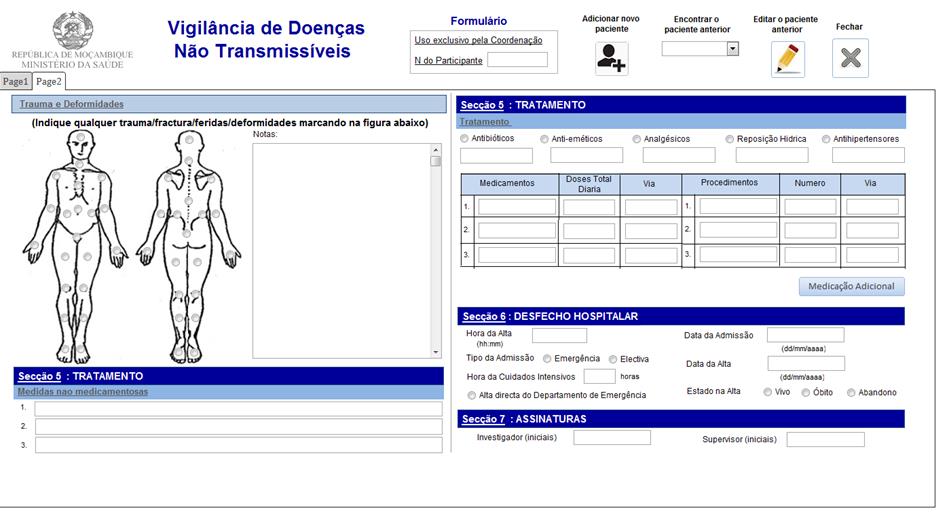
**
